# A pH-Sensing Film from Tamarind Seed Polysaccharide with Litmus Lichen Extract as an Indicator

**DOI:** 10.3390/polym10010013

**Published:** 2017-12-22

**Authors:** Tieqiang Liang, Lijuan Wang

**Affiliations:** 1 Key Laboratory of Bio-Based Materials Science and Technology of Ministry of Education, Northeast Forestry University, Harbin 150040, China; ltq@nefu.edu.cn; 2 Research Center of Wood Bionic Intelligent Science, Northeast Forestry University, Harbin 150040, China

**Keywords:** tamarind seed polysaccharide, pH sensing, litmus lichen extract, packaging film

## Abstract

A new pH-sensing film was developed by using tamarind seed polysaccharide (TSP) and natural dye extracted from litmus lichen (LLE). The addition of LLE from 0 to 2.5% decreased the tensile strength and elongation at break from 30.20 to 29.97 MPa and 69.73% to 60.13%, respectively, but increased the water vapor permeability from 0.399 × 10^−9^ to 0.434 × 10^−9^ g·s^−1^·m^−1^·Pa^−1^. The UV–Vis spectra of the litmus lichen extract (LLE) in the pH range of 4–10 showed that the color clearly changed from orange to blue. The characterization results showed that TSP interacted with LLE through hydrogen bonds. The color of the film varied from orange (pH 4.0) to blue-violet (pH 10.0). The full cream milk spoilage test indicated that the film is suitable for application in full cream milk spoilage detection. The developed pH-sensing film could be used as a promising diagnostic tool for the detection of food spoilage.

## 1. Introduction

**Figure 1:**
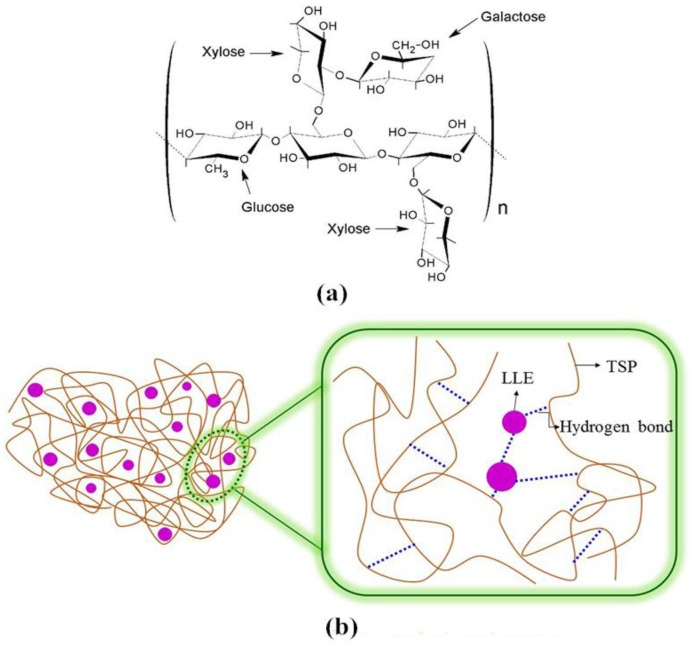
Structure of tamarind seed polysaccharide (TSP) (**a**) and schematic diagram of the TSP film after litmus lichen extract (LLE) addition (**b**).

To reduce white pollution, alternatives to synthetic polymer materials must be explored. In the past years, natural and biodegradable polymers such as chitosan, agar, pectin, and other different polymers have received extensive attention and have been applied as immobilization materials to prepare films [1,2,3]. Among those polymers, polysaccharides have been exploited and applied as immobilization materials owing to their good film-forming and oxygen barrier performances [4,5,6]. Tamarind seed polysaccharide (TSP) is a mucilaginous polysaccharide obtained from the seed kernels of *Tamarindus indica* Linn., which contains glucose, xylose, and galactose units at a ratio of 2.8:2.25:1 [7]. The structure of TSP is shown in Figure 1. It has been reported that TSP is nontoxic and can be used in the food industry [8]. In addition, it has also been employed as a drug-releasing material [9] and in textile printing fields [10]. However, to the best of our knowledge, the use of TSP in films has rarely been reported.

In a previous study, the development of fiber-optic and visual pH sensors was examined because of their compact size, safety, long-distance transmission, sensitivity, and low cost [11]. In the field of food safety, the microbial population and pH of foods have been reported to increase during the storage period owing to protein spoilage, indicating the relationship between the pH of foods and their freshness [12]. Therefore, pH sensing is useful for monitoring or indicating food freshness. Several studies have monitored the pH variations during chilled pork deterioration by using chitosan as an immobilization matrix [13,14]. In general, pH-sensing chemical reagents such as bromocresol compounds, chlorophenol red, and cresol red have been widely used [15,16,17]. However, these synthetic chemical dyes may contaminate the food and pose a threat to human health owing to their potential toxicity [18]. Recently, several studies have reported the potential use of natural pH-sensing dyes such as curcumin and anthocyanin, which are extracted from plants, in intelligent food packaging films [2,12,19,20]. Natural dyes have more obvious advantages, including nontoxicity, easy preparation, renewability, and a non-polluting nature [21]. Many centuries ago, litmus was obtained from lichens by treatment with urine or ammonia, and was widely used for dyeing silk and wool, along with alizarin and indigo. Sometimes, it was also used for dyeing foodstuffs [22]. However, the use of LLE (an extract of litmus lichen), as a pH indicator dye in film has not yet been studied.

In the present study, TSP was used to develop a novel film material, and LLE was used as a natural pH indicator dye, which were both natural biomaterials. The color change of the pH-sensing film was sensitive and could be observed by the naked eye in the pH range of 4.0–10.0. The TSP/LLE films were analyzed by using Fourier transform-infrared spectroscopy (FTIR) and scanning electron microscopy (SEM). In addition, the mechanical properties and color response efficiency of the TSP/LLE films in different buffer solutions at various pH were also tested. Full cream milk was selected as the spoilage material to evaluate the potential use of this film in the food packaging industry, as it represents a mainstream milk product.

## 2. Materials and Methods

### 2.1. Materials

Litmus lichen was purchased from Macklin Biochemical Technology Co., Ltd. (Analytical Reagent, Shanghai, China). TSP was purchased from Shanghai AB Food and Beverages Ltd. (Shanghai, China). Anhydrous glycerin (analytical reagent) was purchased from Yongda Chemical Rregent Co., Ltd. (Tianjin, China). Fresh full cream milk was purchased from the milk industry (Harbin, China).

### 2.2. Experimental Procedures

#### 2.2.1. Extraction Procedure

According to the procedure of the “Pharmacopoeia of People’s Republic of China” with some modifications [23], 0.5 g of litmus lichen powder was added to 200 mL of distilled water and incubated for 1 h at 80 °C with magnetic stirring. Then, the solutions were filtered by using G_3_ sintered discs to remove the solid and other non-hydrophilic materials. The concentration of the LLE solution was 1.49 g/L obtained after the solution was dried to constant weight at 100 °C in a weighted glass dish. 

#### 2.2.2. Development of pH-Sensing Films

A total of 8 g of TSP powder were mixed with 200 mL of distilled water, and the mixture was heated to 90 °C and maintained at this temperature for 2.5 h under constant stirring. The undissolved particles in the solution were removed by centrifugation at 5000 rpm for 10 min [24]. Then, glycerol was added to the solution at a concentration of 35 g/100 g TSP and stirred for 30 min. Subsequently, LLE solution was added and the LLE content reached 1.25 g/100 g and 2.5 g/100 g (TSP basis), labeled as LLE-1.25 and LLE-2.5, respectively. In order to reduce the break to the structure of LLE and prevent the deterioration of TSP (according to our pre-experiment, a drying temperature below 80 °C can cause the deterioration of TSP), the resulting solution was cast into a glass plate (26 cm × 26 cm × 4 cm) after removing bubbles and dried for 10 h at 80 °C in a vacuum oven.

### 2.3. Characterization

#### 2.3.1. Mechanical Properties

The thickness of the films was measured with an ID-C112XBS micrometer (Mitutoyo Corp., Tokyo, Japan), and recorded as the average of values measured at five random points. Tensile tests were performed using an auto tensile tester (XLW-PC, PARAM, Jinan, China) equipped with a 500 N load cell under a strain rate of 300 mm/min at 25 °C [25].

#### 2.3.2. Water Vapor Permeability

The water vapor permeability (WVP) of the films was measured according to our previous study [6]. The cups, which contained anhydrous calcium chloride desiccant, were covered with the developed films. The mouths of the cups were sealed and the test assemblies were incubated at 25 °C and 75% relative humidity (saturated NaCl solution). The driving force was 1753.55 Pa (Δ*P*), which was expressed as water vapor partial pressure. The cups were weighed periodically to determine the amount of moisture transferred through the sample into the desiccant. WVP was realized in the steady state of the weight versus time result, and calculated by dividing the slope of the line by the exposed film area. Each film was tested in triplicate.

#### 2.3.3. UV–Vis Spectroscopy

The UV–vis spectra of the LLE solutions were measured by using a UV–vis spectrophotometer (UV-2600, Shimadzu, Kyoto, Japan) at 400–700 nm. Before the measurement of the spectra of the LLE solutions (pH 4.0–10.0), the pH of the solutions were adjusted to the desired level by adding 0.1 M HCl or NaOH.

#### 2.3.4. FTIR Spectroscopy

FTIR analysis was performed using a Nicolet 6700 spectrometer (Thermo Fisher Scientific, Waltham, MA, USA), with an attenuated reflectance (ATR) Fourier transform mode. The FTIR spectra of the films were measured between 4000 and 500 cm^−1^ and recorded at a 4 cm^−1^ resolution.

#### 2.3.5. SEM Observation

Micrographs of the films were observed by using a Quanta 200 scanning electron microscope (Philips-FEI Co., Eindhoven, The Netherlands) under an accelerating voltage of 5 kV after applying a thin gold layer onto the films prior to observation.

#### 2.3.6. Color Response Analysis

The color changes of the pH-sensing films were measured by using a colorimeter (CM–2600d, Konica Minolta, Tokyo, Japan). Before the measurement, the pH-sensing films were immersed in 5 mL of each buffer solution at pH 4.0–10.0 for 5 s. After removing the buffer solutions, each film was placed on a white plate and its color was noted before drying. The results were expressed as L* (lightness), a* (red to green), and b* (yellow to blue) parameters to evaluate the color changes in different buffer solutions at various pH levels. All of the measurements were determined at three random points on both sides of each film, and the experiments were performed in triplicate.

#### 2.3.7. Application of the Developed Film in the Detection of Full Cream Milk Spoilage

Full cream milk spoilage is related to its pH, acidity, and microbial load, among which the acidity of the milk is the most important index [26]. In the present study, the acidity of milk was initially measured by the titration method (according to Chinese GB/T 5009.46-2003). In brief, 10 mL of milk and 20 mL of sterile purified water were titrated against 0.1 mol/L NaOH using alcoholic phenolphthalein as an indicator at 40 °C. An acidity between 16 and 18 °T revealed that the milk was fresh, that between 18 and 20 °T showed that the milk was suitable for consumption, and that beyond 20 °T indicated that the milk was spoiled and could not be consumed. Meanwhile, the color changes of the pH-sensing film in milk with different acidity levels were tested after the pH-sensing film was immersed in 5 mL of milk with different acidity levels by using a colorimeter (CM–2600d, Konica Minolta, Tokyo, Japan).

#### 2.3.8. Statistical Analysis

The data obtained were analyzed by analysis of variance using SPSS (version 17.0, SPSS Inc., Chicago, IL, USA). Duncan’s multiple-range test (*p* < 0.05) was used to compare the differences between the films’ properties.

## 3. Results and Discussion

### 3.1. Mechanical Properties of the pH-Sensing Films

The thickness, tensile strength (TS), and elongation at break (EAB) of the TSP films with various concentrations of LLE are shown in Table 1. There was no significant change in the thickness of the films. However, with the increasing concentration of LLE up to 2.5%, the TS and EAB of the films decreased from 30.20 to 29.97 MPa and 69.74% to 60.13%, respectively, owing to the decrease in intramolecular and intermolecular interaction between the TSP molecules and plasticizers, which are correlated to the LLE structure and composition. In particular, the EAB value significantly decreased. These results corresponded to those obtained in the FTIR analysis.

### 3.2. WVP of the pH-Sensing Films

As shown in Table 1, the WVP of the pH-sensing films gradually increased from 0.399 × 10^−9^ to 0.434 × 10^−9^ g·s^−1^·m^−1^·Pa^−1^ with the increase in the LLE content from 0 to 2.5 g/100 g TSP, but the WVP values of films were still very low. The increase of WVP values may be owing to the fact that LLE is hydrophilic and the addition of LLE caused a decrease in the intramolecular and intermolecular interactions.

### 3.3. UV–Vis Spectroscopy of LLE Solutions at Various pH Ranges

**Figure 2:**
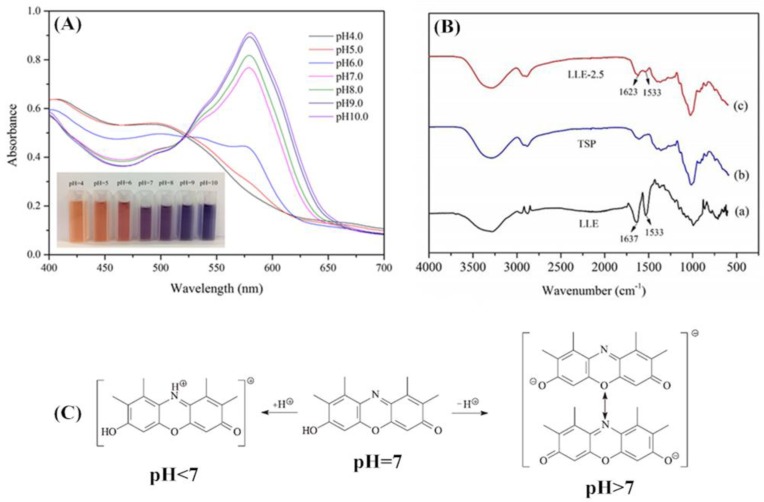
(**A**): UV–Vis spectra of the LLE at different pH values; (**B**): FTIR spectra of LLE (a), TSP film (b), and pH-sensing film (c); (**C**): the detail mechanism of LLE at different pH levels [22].

The color variations of the diluted LLE solutions were examined and recorded to validate its use as a pH-sensing dye. The initial pH of the LLE solution was 10.0. Figure 2A shows that the color of the LLE solution changed from blue to purple, orange, and yellow when the pH was 10.0–9.0, 8.0–7.0, 6.0–5.0, and 4.0, respectively. In general, visible color absorbs light of wavelengths corresponding to its complementary color [20], and orange and blue are complementary colors [27]. The maximum absorption peak at 580 shifted to 576 nm when the pH decreased from 10.0 to 6.0, and the strength of the peak also decreased. When the pH was <6.0, the peak disappeared, which resulted in the change in the color of the LLE solution from blue to orange. Moreover, the maximum absorption peak at 495 shifted to 580 nm when the pH increased from 4.0 to 10.0, and the strength of the peak at 495 decreased while the strength of the peak at 580 increased. The adsorption peaks around 495 and 580 nm were due to the presence of hydroxyorceins, and the 7-hydroxyphenoxazone chromophore was the principal constituent. The adsorption peak change was related to the change in chemical structure. In an acidic condition, a proton combined with the nitrogen atom in 7-hydroxyphenoxazone chromophore to obtain the red cation, while above pH 7, 7-hydroxyphenoxazone chromophore eliminated a proton to give the mesomeric to form a blue-violet anion; detail mechanism is shown in Figure 2C [22,28,29]. As a result, the absorption peak shifted to longer wavelengths with the pH increasing from 4.0 to 10.0.

### 3.4. FTIR Spectra of the pH-Sensing Films

The FTIR spectra of LLE, TSP film, and the pH-sensing film (LLE-2.5) are shown in Figure 2B. As indicated in Figure 2Ba, the bands at around 3400, 1637, 1533, and 1000 cm^−1^ correspond to –OH stretching vibration, C=C stretching vibration, N–H bending vibration, and C–O stretching vibration, respectively [30,31]. Furthermore, the bands at 2941 and 2879 cm^−1^ can be attributed to –CH in-plane deformation vibration. These comprehensive spectral patterns were noted to be in accordance with the appropriate results with featured molecular frames extracted from litmus lichen [22]. 

Figure 2Bb indicates that a broad band at around 3400–3200 cm^−1^ corresponds to –OH stretching vibrations [32]. The bands at 2940 and 2888 cm^−1^ can be attributed to the feature of glycerol, and the band at 1618 cm^−1^ corresponding to N–H bending vibration may indicate the presence of residual proteins [33]. In particular, the bands at 1022, 1153, 1618, 2888, and 3300 cm^−1^ can be attributed to –H=C stretching vibration, –C–O–C– asymmetric stretching vibration of glucopyranosyl and xylopyranosyl units, –CH–OH stretching vibration, aliphatic C–H stretching vibration, and –OH stretching vibration, respectively [34]. In addition, the bands at 930–730 cm^−1^ indicate the vibration of pyranose rings in polysaccharides [35].

As illustrated in Figure 2Bc, an increase in the intensity of band at 1623 cm^−1^ and a new band at 1533 cm^−1^ in the LLE pH-sensing film spectrum could be attributed to the addition of LLE. Moreover, it can be noted that the bands at 1022 and 1618 cm^−1^ observed in Figure 2Bb moved to a higher wavelength (1027 and 1623 cm^−1^), suggesting that the addition of LLE reduced the interactions among TSP molecules in the film [36]. These results showed that LLE was immobilized onto the TSP film, and that the addition of LLE produced weak intermolecular interactions in the film. The concise structural model of the TSP film after LLE addition is shown in Figure 1.

### 3.5. SEM Observation

**Figure 3:**
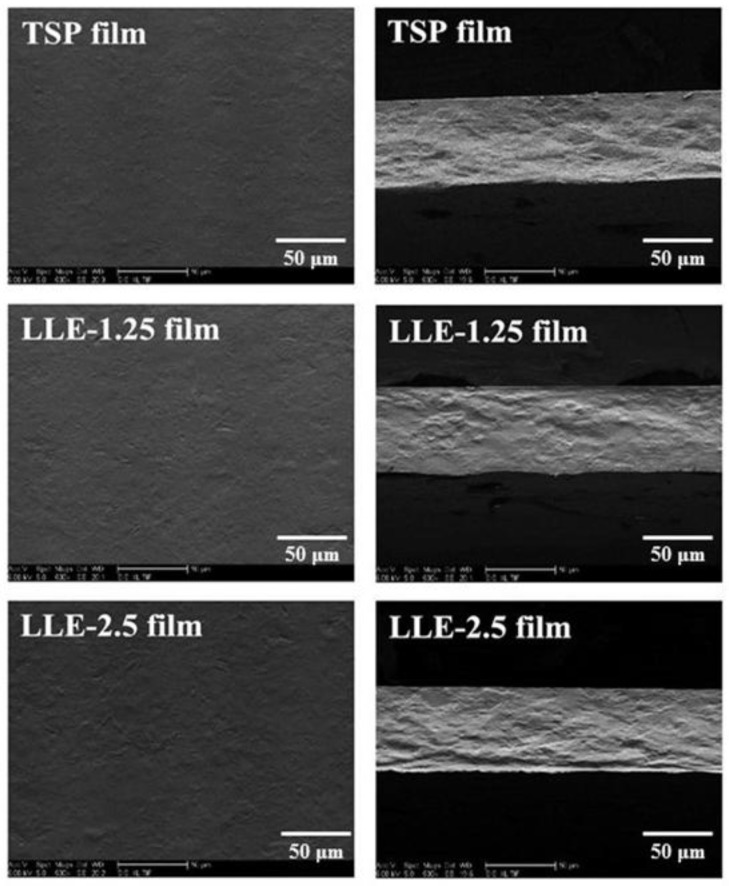
SEM of the TSP film and pH-sensing film (LLE-2.5). Left micrograph shows the surface of the film and right micrograph illustrates the cross-section of the film.

The micrographs of the surfaces and freeze-fractured cross-sections of the TSP film and pH-sensing film (LLE-1.25 and LLE-2.5) are presented in Figure 3. In the absence of LLE, the surface of the TSP film was slightly rough, because TSP is a polyhedral structure [7]. The roughness of the film increased with the addition of LLE owing to the reduced intermolecular interactions between the TSP molecules.

### 3.6. Color Response Analysis of the pH-Sensing Films

According to the results of the pre-experiment, LLE-2.5 was selected for the color response test. The color response of the pH-sensing film was evaluated by immersing the film into different buffer solutions (pH 4.0–10.0) for 5 s. Table 2 shows the visible color changes of each sample from orange to purple in different buffer solutions (pH 4.0–10.0). The values of a* and b* parameters were significant. With the decrease in the a* and b* values, the pH-sensing film turned bright orange, fuchsia, purple, and blue-violet at pH 4.0, 5.0–6.0, 7.0–8.0, and 9.0–10.0, respectively, indicating that the film is sensitive to pH.

### 3.7. Full Cream Milk Spoilage Detection by the pH-Sensing Films

To validate the reliability of the use of pH-sensing films in practical applications, the acidity of milk was detected by using the pH-sensing films (Table 3). The initial acidity of milk was 16.35 °T, which indicated that the milk was fresh. After 7 h, the acidity increased to 18.03 °T, which suggested the start of milk spoilage. In 8 h, the milk was significantly spoiled owing to the acute increase in lactic acid concentration, and the pH of the milk obviously changed. With regard to the color parameters, there was only a slight difference in the L* value, whereas the a* and b* values increased with the decreasing pH and increasing acidity, and the pH-sensing films turned to red and yellow. After 8 h, the color of the film turned from blue-violet to fuchsia, and after 10 h, the film turned from blue-violet to orange, indicating that the milk was completely spoiled. These results showed that the pH-sensing film could detect milk spoilage and can be used in practical applications. 

## 4. Conclusions

In this study, natural dye extracted from litmus lichen was used as a pH indicator, and a bio-based material, TSP, was employed to immobilize the natural dye to prepare a pH-sensing film. The addition of LLE from 0 to 2.5% TSP decreased the TS and EAB from 30.20 to 29.97 MPa and 69.73% to 60.13%, respectively, while it increased the WVP from 0.399 × 10^−9^ to 0.434 × 10^−9^ g·s^−1^·m^−1^·Pa^−1^. The color of the film varied from yellow to blue in the pH range of 4.0–10.0. FTIR spectra indicated the presence of hydrogen bonds between the polymers. While the addition of LLE interrupted the intermolecular and intramolecular interactions, which decreased the TS and EAB of the TSP film, it enhanced the WVP, although the WVP values were still very low. The milk spoilage test showed that the pH-sensing film could detect the spoilage of milk and can have practical applications. The developed pH-sensing film exhibited good responses to pH change in solutions, which suggested its significant potential as an intelligent package material in the food industry to monitor the freshness of packaged foods. Further studies will be conducted to determine the correlation between the color of the pH-sensing film and various foods to estimate the degree of spoilage of foods.

## Figures and Tables

**Table 1:** Mechanical properties and water vapor permeability (WVP) values of the pH-sensing film.

Sample	Thickness (mm)	TS (MPa)	EAB (%)	WVP (g·s^−1^·m^−1^·Pa^−1^ × 10^−9^)
TSP film	0.082 ± 0.011 ^b^	30.20 ± 2.62 ^a^	69.73 ± 5.20 ^a^	0.399 ± 0.061 ^a^
LLE-1.25	0.077 ± 0.003 ^a,b^	30.16 ± 0.24 ^a^	68.13 ± 5.91 ^a^	0.400 ± 0.060 ^a^
LLE-2.5	0.076 ± 0.003 ^a^	29.97 ± 1.25 ^a^	60.13 ± 1.42 ^a^	0.434 ± 0.087 ^b^

^a,b^ signify that different superscripts in the same parameters indicate significant differences (*p* < 0.05).

**Table 2:** Color parameters of the pH-sensing film in different buffer solutions at pH 4.0–10.0.

pH Value	L*	a*	b*	Pictures
4.0	64.39 ± 0.47 ^c^	15.45 ± 0.26 ^g^	8.22 ± 0.28 ^e^	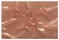
5.0	61.87 ± 0.78 ^b^	12.23 ± 0.45 ^f^	2.24 ± 0.37 ^d^	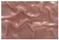
6.0	61.88 ± 1.04 ^b^	9.66 ± 0.38 ^e^	−1.27 ± 0.70 ^c^	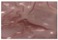
7.0	59.67 ± 1.40 ^a,b^	5.48 ± 0.29 ^d^	−6.37 ± 0.23 ^b^	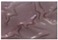
8.0	61.76 ± 1.57 ^a,b^	4.06 ± 0.21 ^c^	−7.24 ± 1.12 ^a,b^	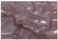
9.0	59.59 ± 1.53 ^a^	2.43 ± 0.04 ^a^	−7.45 ± 0.03 ^a^	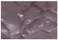
10.0	60.74 ± 1.17 ^a,b^	3.11 ± 0.17 ^b^	−8.11 ± 0.11 ^a^	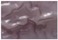

^a–g^ signify that different superscripts in the same parameters indicate significant differences (*p* < 0.05).

**Table 3:** Color parameters, pH value, and acidity of the pH-sensing film immersed in milk at different time points.

Time (h)	L*	a*	b*	pH Value	Acidity (°T)
0	51.33 ± 0.517 ^a^	9.22 ± 0.252 ^a^	−4.13 ± 0.229 ^a^	6.78	16.35
6	54.10 ± 0.907 ^a^	9.38 ± 0.283 ^a^	−3.64 ± 0.092 ^a,b^	6.62	16.36
7	55.03 ± 1.175 ^a^	10.04 ± 0.670 ^a,b^	−3.38 ± 0.303 ^b^	6.54	18.03
8	53.82 ± 0.664 ^a^	10.48 ± 0.014 ^b,c^	−3.08 ± 0.140 ^b^	6.32	20.55
9	50.92 ± 0.573 ^a^	11.21 ± 0.417 ^c^	−1.01 ± 0.205 ^c^	5.98	27.26
10	54.11 ± 2.833 ^a^	11.35 ± 0.295 ^c^	−0.82 ± 0.365 ^c^	5.49	35.23

^a–c^ signify that different superscripts in the same parameters indicate significant differences (*p* < 0.05).
